# Correlation of PK/PD Indices with Resistance Selection for Cefquinome against *Staphylococcus aureus* in an *In Vitro* Model

**DOI:** 10.3389/fmicb.2016.00466

**Published:** 2016-04-12

**Authors:** Yafei Li, Baoyi Feng, Xiaoyan Gu, Dawei Yang, Zhenling Zeng, Bingxu Zhang, Huanzhong Ding

**Affiliations:** ^1^Laboratory of Veterinary Pharmacology, College of Veterinary Medicine, South China Agricultural UniversityGuangzhou, China; ^2^Centre for Veterinary Drug Residues, College of Veterinary Medicine, South China Agricultural UniversityGuangzhou, China; ^3^China Institute of Veterinary Drug ControlBeijing, China

**Keywords:** cefquinome, *Staphylococcus aureus*, PK/PD, MPC, *in vitro* model

## Abstract

Cefquinome is a fourth-generation Cephalosporin approved for use in animals exclusively. The objective of this study was to explore the relationship of cefquinome pharmacokinetic/pharmacodynamic (PK/PD) indices with resistance selection of *Staphylococcus aureus* ATCC25923 in an *in vitro* model. Six dosing regiments of cefquinome at an interval of 24 h for three consecutive times were simulated, resulting in maximum concentrations (C_max_) from 1/2 to 16MIC and terminal half-lives (t_1/2β_) of 3 and 6 h, respectively. The *in vitro* sensitivity of *S. aureus* was monitored by bacterial susceptibility and dynamic time-kill curve experiments over the six cefquinome concentrations. The correlation between changes in bacterial susceptibility (MIC_72_/MIC_0_) and the percentage of time within mutant selection window versus dosing interval (T_MSW_ %) was subjected to the Gaussian function and regression analysis. Our results favored the consensus that time above MIC (T > MIC) was recognized as an important PK/PD parameter of cephalosporins for antibacterial efficiency. Cefquinome reached the maximum killing effect when T > MIC% attained approximately 40∼60%. The subsequent correlation analysis demonstrated that resistant *S. aureus* ATCC25923 was easy to occur when T_MSW_% attained an index of about 20% with t_1/2β_ of 3 h after multiple dosing, and 40% with t_1/2β_ of 6 h after multiple dosing, respectively.

## Introduction

*Staphylococcus aureus* is a major pathogen for animals and humans, which contributes to a variety of severe infections, including bacteremia, meningitis, endocarditis, skin and wood infection or other diseases for animals and humans (Archer, 1998; Lowy, 1998; Azizoglu et al., 2013). More importantly, *S. aureus* plays an important role in the food contamination by foodborne pathogens. Food poisoning caused by staphylococcal enterotoxin (SE) is a pressing worldwide health problem (Tauxe, 2002; Le Loir et al., 2003). In spite of the progress in antimicrobial therapy, treatment of *S. aureus* infection has become more and more challenging because drug-resistance of *S. aureus* has increased globally over the past decade. The methicillin-resistant *S. aureus* (MRSA) and vancomycin-resistant *S. aureus* (VRSA) were particularly reported and well documented (Harris et al., 2010; Mohammed Fayaz et al., 2011).

In veterinary medicine, cefquinome (CEQ) has been licensed in European countries by virtue of its broad antibacterial spectrums and remarkable antibacterial activities. Despite of this, the cephalosporins should be in prudent use considering the escalating antimicrobial resistance. Evidence has showed that different animal species may harbor the same resistance determinant and are recognized as possible reservoirs of antimicrobial-resistant bacteria (Guardabassi et al., 2004). It has been also observed that cefquinome exerted a selective effect on *bla*_CTX-M_ producing *Escherichia coli* strains (Cavaco et al., 2008). Therefore, further information concerning the ability of cefquinome to prevent occurring of resistant strains seems to be investigated.

Since mutant prevention concentration (MPC) of antibiotics was first described by Dong et al. (1999), it has been successfully applied to evaluate the ability of antibiotics that restrict the selection of resistant strains (Allen et al., 2004; Allen and Hankins, 2009; Wang et al., 2010; Blondeau et al., 2012) and optimize the current dosing regimens of antibiotics to slow the emergence of resistant strains. MPC was taken as the lowest doubling dilution drug concentration that prevented a population of 10^10^ colony forming unit (CFU)/mL or even more microorganisms from first-step mutation. Zone of the drug concentrations between minimum inhibitory concentration (MIC) and MPC was defined as the mutant selection window (MSW). MPC and MSW concepts, representing the ability of antibiotics to select resistant strains, have been tested in various *in vitro* studies (Allen et al., 2004; Croisier et al., 2004b; Ferran et al., 2007), in *ex vivo* pharmacodynamic studies (Bronner et al., 2002) and in *in vivo* studies (Croisier et al., 2004a; Cui et al., 2006). Here, we developed an *in vitro* kinetic model to investigate the relationship between pharmacokinetic/pharmacodynamic (PK/PD) indices of cefquinome with terminal half-lives (t_1/2β_) of 3 or 6 h and resistance development of *S. aureus* ATCC25923.

## Materials and Methods

### Antimicrobial Agents, Medium, and Bacterial Strains

Raw material of cefquinome (purity of 84.1%) was obtained from Hebei yuanzheng Pharmaceutical Enterprise, Co., Ltd. Mueller-Hinton broth (MHB) and agar were purchased from Guangzhou huankai Comapany. *S. aureus* ATCC 25923 was purchased from the China Institute of Veterinary Drug Control (Beijing, China).

### *In Vitro* Susceptibility Testing

The MIC of CEQ against *S. aureus* with an inoculum of 5 × 10^5^ CFU/mL was determined by standard agar dilution method established by the Clinical and Laboratory Standards Institute (CLSI).

### Measurement of MIC_99_, Mutant Prevention Concentration (MPC) and Selection Index (SI)

The MIC_99_ was defined as the drug concentration that inhibited 99% of bacteria colony formation. MPC was reckoned as the lowest cefquinome concentration blocking ≥10^10^ CFU/mL inoculants growth. The measurements of MIC_99_ and MPC were mainly based on the method reported by Zhao and Drlica (2002) with slight modification. For MIC_99_, agar plates containing a series of cefquinome (concentrations ranging from 0.5 to 0.164 μg/mL) at 20% per sequential decrease were prepared. 3 × 10^7^ CFU/mL *S. aureus* suspension in logarithmic phase of growth was then subjected to serial 10-fold dilutions with MHB to 3 × 10^2^ CFU/mL bacteria. Again, 100 μL of each dilution was plated onto the agar plates containing the series of CEQ concentrations mentioned above and incubated at 37°C overnight. Bacterial colonies that recovered growth in each dilution were counted. Drug concentrations versus the percentages of colony recovery were plotted and the interpolation method was adopted to calculate the cefquinome concentration blocking 99% bacteria growth.

The determination of MPC was similar to that of MIC_99_. Instead, 100 μL of 10^10^ CFU/mL inoculants was spread on agar plates containing a series of antimicrobial concentrations. The lowest antimicrobial concentration preventing bacterial colony formation at 72 h after incubation was measured as provisional mutant prevention concentration (MPC_pr_). A second determination that utilized linear MPC_pr_ decreases (about 20% per sequential decrease) was performed. MPC was the lowest CEQ concentration preventing 100% growth of *S. aureus* colony.

The calculated ratio of MPC/MIC_99_ for *S. aureus* was defined as selection index (SI) of cefquinome.

### *In Vitro* Dynamic PK/PD Model Simulation

**FIGURE 1:**
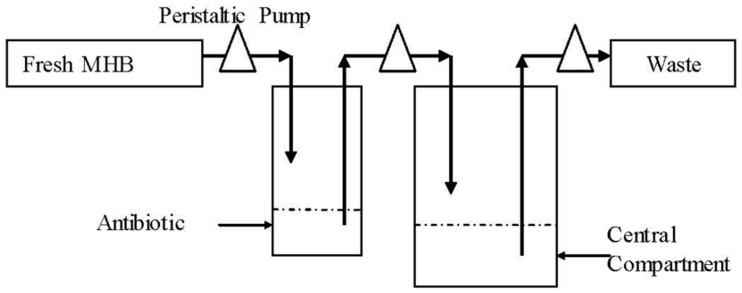
**Scheme of the *in vitro* dynamic model for bactericidal kinetics**.

A previous described dynamic model (Grasso et al., 1978) with modification was developed in this study. One-compartment open model with first-order absorption of pharmacokinetics for cefquinome was simulated. The schematic representation of *in vitro* PK/PD model used is depicted in **Figure 1**. Briefly, the system was mainly composed of three sealed containers (compartments) and they were connected with peristaltic pumps in line, each containing sterile MHB and a magnetic stirrer to ensure adequate mixing. One 500 mL sealed compartment containing 300 mL MHB, provided with either a bacterial culture alone (control growth experiments) or a bacterial culture plus antibiotic (killing/re-growth experiments), acted as the central chamber. Another 100 mL sealed container charged with 60 mL of sterile MHB, acted as the absorption chamber, with desired calculated drug concentrations. The third one was used to provide fresh MHB. Waste was also collected. Peristaltic pumps circulated fresh broth to central and absorption compartments at the desired flow rate. The flow rate of the pump was set on the basis of the terminal half-life being simulated. A series of dosage regimens were designed to generate different CEQ concentration profiles with terminal half-lives of 3 and 6 h for three consecutive administrations in this apparatus.

### *In Vitro* PK/PD Model and CEQ Dosing Regimens

The apparatus ran under 37°C. The elimination terminal half-lives of cefquinome reported in the literature varied from 0.5 to 10 h (Limbert et al., 1991; Li et al., 2008; Al-Taher, 2010; Tohamy, 2011; Yuan et al., 2011; Zonca et al., 2011; Dumka et al., 2013), therefore, 3 and 6 h were selected in our study. Peristaltic pumps circulated fresh MHB medium to and from the central compartment at a flow rate of 15.75 r/min for t_1/2β_ of 3 h or 7.86 r/min for t_1/2β_ of 6 h, respectively. 3 mL bacterial suspensions (10^8^ CFU/mL) at the logarithmic phase were injected into the central compartment container. The bacteria were incubated in the model for 2 h to result in exponentially growing cultures before addition of cefquinome. Antibiotic doses were calculated to generate CEQ initial concentrations of 1/2MIC, MIC, 2MIC, 4MIC, 8MIC, and 16MIC levels, respectively. The *in vitro* pharmacokinetics of cefquinome for all doses after three consecutive administrations with 24 h interval for terminal half-lives of 3 and 6 h were simulated.

### Quantification of Cefquinome in MHB

Samples were collected from the central compartment immediately before and at 0.5, 1.0, 2.0, 3.0, 4.0, 6.0, 8.0, 10.0, 12.0, and 24.0 h after the first and second administration, and 0.5, 1.0, 2.0, 3.0, 4.0, 6.0, 8.0, 10.0, 12.0, 24.0, 30.0, 36.0, and 48.0 h after the third administration. These samples were stored at -20°C until analysis.

1.5 mL of MHB was extracted for the determination of cefquinome. The concentrations of cefquinome in MHB were analyzed by high-performance liquid chromatography-MS/MS (HPLC-MS/MS) with an assay range of 0.005–0.5 μg/mL. The protein in MHB sample (1 mL) was precipitated by 2 mL acetonitrile, and the supernatant was directly injected into HPLC-MS/MS after high speed centrifugation. Analysis of quality control (QC) samples at three levels (0.01, 0.05, 0.2 μg/mL) showed that the recoveries of the method were above 70%; the intra-day and inter-day coefficients of variation were within 15%. The pharmacokinetic parameters were calculated by a WinNonlin software (version 5.2.1; Pharsight Corporation, USA).

### *In Vitro* Time-Kill Experiments and Susceptibility Testing of *S. aureus*

To measure the antimicrobial effect of cefquinome with different terminal half-lives against *S. aureus*, the colony count and susceptibility of bacteria in each time point were performed after treatment. Half of each sample was subjected to time-kill kinetic assays. Samples (100 μL) in series of 10-fold dilution with sterile saline (0.9% NaCl) were spread onto Mueller-Hinton agar to determine the number of total or resistant cells. The log_10_ of surviving *S. aureus* cells (CFU/mL) was plotted against each time point. The other half of each sample was used for the susceptibility test. MIC values of bacteria post-exposed in each time point was conducted using tube dilution method according to the criteria established by the Clinical Laboratory Standards Institute [CLSI] (2013).

### Pharmacokinetic/Pharmacodynamic (PK/PD) Integration Analysis

By using the individual pharmacokinetic result from each *in vitro* dosage regimen, the following PK/PD parameters were obtained: ratio of area under the curve of cefquinome concentration versus time to MIC (AUC_0-∞_/MIC) or (AUC_0-24__h_/MIC), ratio of area under the curve of cefquinome concentration exceeding MIC to MIC (AUC_C__>__MIC_/MIC), time of concentration above MIC (T > MIC) or MPC (T > MPC), time of concentration within MPC and MIC (T_MSW_) or expressed as percentage (T_MSW_%), ratio of MIC_72_ to MIC_initial_ (MIC_72_/MIC_initial_).

Gaussian function was used to simulate the correlation of MIC_72__h_/MIC_initial_ with T_MSW_% (percentage of the time during which cefquinome concentrations were inside the MSW). The formula was as follows:

$$y=y_{0}+\frac{A}{\sqrt{2\,\pi \,\sigma }}\,e^{-\frac{{\left(x-x_{0}\right)}^{2}}{2\,\sigma^{2}}}$$

where A was the area under curve and upper baseline; x_0_ was the minimum value of T_MSW_% which resulted in the maximum MIC_72__h_/MIC_initial_.

## Results

### MIC_99_ and MPC

The MIC_99_ and MPC of cefquinome against *S. aureus* ATCC 25923 were 0.4 and 4.096 μg/mL, respectively. So the SI was 10.24 (MPC/MIC_99_).

### *In Vitro* Simulated Pharmacokinetics

**FIGURE 2:**
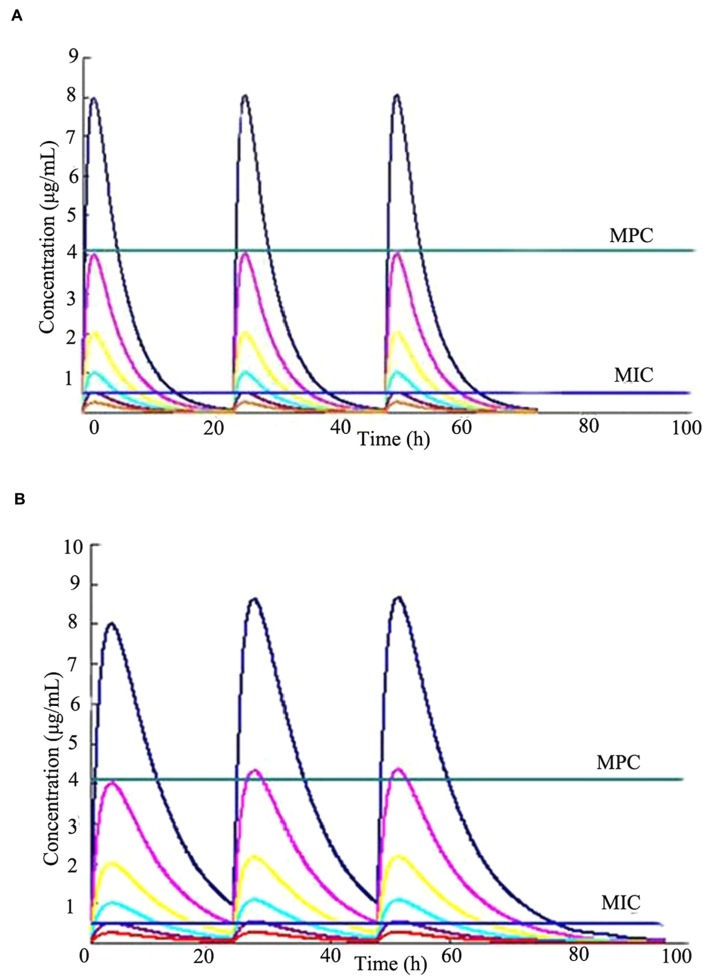
**Concentration-time curves of cefquinome in the *in vitro* pharmacokinetics model of two terminal half-lives (t_1/2β_).** The horizontal lines indicate the MPC (4.096 μg/mL) and MIC (0.4 μg/mL) for *Staphylococcus aureus* ATCC 25923, respectively. **(A)** t_1/2β_ = 3 h, **(B)** t_1/2β_ = 6 h.

The *in vitro* simulated time-concentration curves for cefquinome with terminal half-lives of 3 and 6 h are shown in **Figure 2**. This study used time-concentration curves of the unbound fractions of cefquinome in Mueller-Hinton broth. The maximum concentrations were approximately equal to 1/2MIC to 16-fold MIC of cefquinome against *S. aureus* ATCC 25923 after three consecutive administrations. The MPC and MIC levels were also indicated in the simulated time-concentration curves.

### The Bacteria Killing Curves in *In Vitro* Models

**FIGURE 3:**
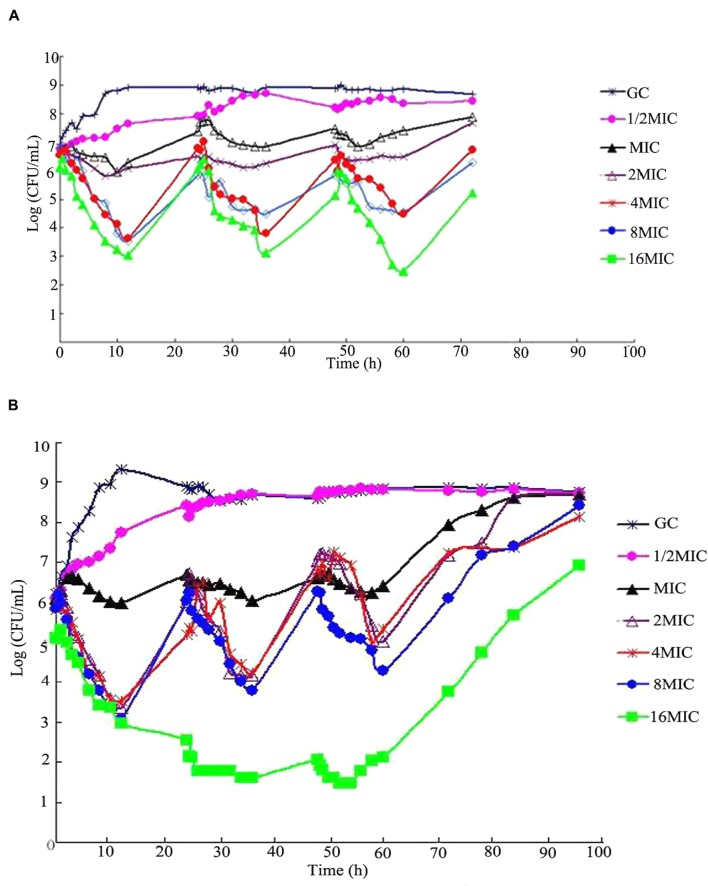
**Viable counts of *S. aureus* ATCCC 25923 in time-kill assays with cefquinome in different terminal half-lives (t_1/2β_) at cefquinome concentrations from a half to 16-fold the minimum inhibitory concentration (MIC), in comparison with a cefquinome-free control. (A)** t_1/2β_ = 3 h, **(B)** t_1/2β_ = 6 h.

Time-killing curves of *S. aureus* ATCC 25923 in the *in vitro* model under different dosing regimens are shown in the following **Figure 3**. For a t_1/2β_ of 3 h multiple dosages, bacteriostatic or bactericidal action was observed when maximum cefquinome concentrations were equal to MIC or over MIC levels (dosages ranged from 0.672 to 10.767 mg). For a t_1/2β_ of 6 h multiple dosages, continuous bactericidal action was observed when maximum cefquinome concentrations were at 4MIC, 8MIC, 16MIC (dose ranged from 2.691 to 10.767 mg), and the continuous time of bactericidal action at 16MIC was the longest among three concentrations.

### Loss of Susceptibility to Cefquinome

**FIGURE 4:**
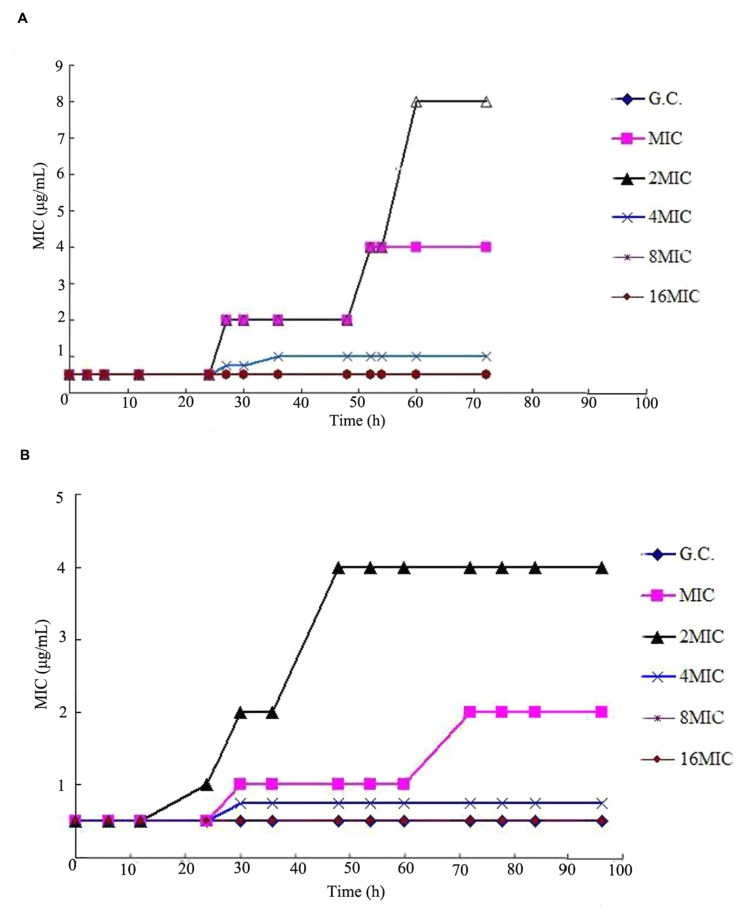
**Typical susceptibility responses of *S. aureus* ATCCC 25923 observed in the *in vitro* PK/PD model simulating the pharmacokinetics of cefquinome with different terminal half-lives (t_1/2β_).(A)** t_1/2β_ = 3 h, **(B)** t_1/2β_ = 6 h.

Compared to drug-free group, loss of susceptibility was observed in MIC, 2MIC, and 4MIC concentration groups after administrations, as shown in **Figure 4**. For multiple dosages with t_1/2β_ of 3 h, the bacterial MICs after administration increased to 4.0, 8.0, and 1.0 μg/mL for MIC, 2MIC, and 4MIC groups, respectively. For multiple dosages with t_1/2β_ of 6 h, the bacterial MICs after administration increased to 2.0, 4.0, and 0.8 μg/mL for MIC, and 2MIC, and 4MIC concentration administrations, respectively. The MIC values did not change through the whole experiment period in 8MIC and 16MIC administration groups with two different terminal half-lives.

### Correlation of PK/PD Indices with Resistance Selection

Pharmacokinetic/pharmacodynamic indices, such as AUC_24__h_/MIC (where AUC_24h_ is the area under the drug concentration time curve in a 24 h interval) and time above the MIC, provide an empirical way to relate antimicrobial dose to favor the treatment effect of bactericidal agents. Relationships between PK/PD indices and loss of susceptibility are shown in **Table 1**. For cephalosporin, T > MIC was the index most commonly associated with restricting susceptible cell growth. When T > MIC attained 17.14 h or T > MIC% attained 24%, the MIC value of cefquinome against *S. aureus* increased to 16-fold of initial value at 72 h after multiple administrations in groups with t_1/2β_ of 3 h. Similarly, in groups with t_1/2β_ of 6 h, when T > MIC = 34.28 h or T > MIC% = 48%, the value of cefquinome MIC increased to eightfold of initial value after multiple dosages.

**Table 1:** Pharmacokinetic/pharmacodynamic (PK/PD) indices of antimicrobial efficacy and risk of resistance selection amongst *Staphylococcus aureus* ATCC 25923 over the complete dosing interval following PK simulations of three consecutive administrations of cefquinome in t_1/2β_ of 3 and 6 h.

t_1/2β_ (h)	D (mg)	$\frac{A\,U\,C_{C>M\,I\,C}}{M\,I\,C}\,\left(h\right)$	$\frac{A\,U\,C_{24}}{M\,I\,C}\,\;\left(h\right)$	T > MIC (h)	T > MPC (h)	T_MSW_ (h)	T_MSW_%	T > MIC%	$\frac{M\,I\,C_{72}}{M\,I\,C_{i\,n\,i\,t\,i\,a\,l}}$
3	1/2MIC	0	3.22	0	0	0	0	0	1
	MIC	6.41	6.43	8.11	0	8.11	11	11	8
	2MIC	25.89	12.89	17.14	0	17.14	24	24	16
	4MIC	64.68	25.76	26.13	0	26.13	36	36	2
	8MIC	142.34	51.52	35.13	7.81	27.32	38	49	1
	16MIC	297.75	103.07	44.14	16.82	27.32	38	61	1

6	1/2MIC	0	5.96	0	0	0	0	0	1
	MIC	12.81	11.92	16.21	0	16.21	23	23	4
	2MIC	51.77	23.89	34.28	0	34.28	48	48	8
	4MIC	129.35	47.73	52.26	0	52.26	73	73	1.5
	8MIC	284.68	95.45	70.26	15.63	54.64	76	98	1
	16MIC	595.50	190.96	88.28	33.64	54.64	76	123	1

Other PK/PD indices also showed correlation with the selection of resistance (**Table 1**). When T_MSW_ and T_MSW_% were 17.14 h and 24%, the MIC increased to 16-fold of initial value in groups with t_1/2β_ of 3 h after multiple dosages, and the MIC increased to eightfold of initial value When T_MSW_ and T_MSW_% were 34.28 h and 48% with t_1/2β_ of 6 h after multiple dosages, respectively.

### Correlation Analysis of MIC Increase with T_MSW_%

**FIGURE 5:**
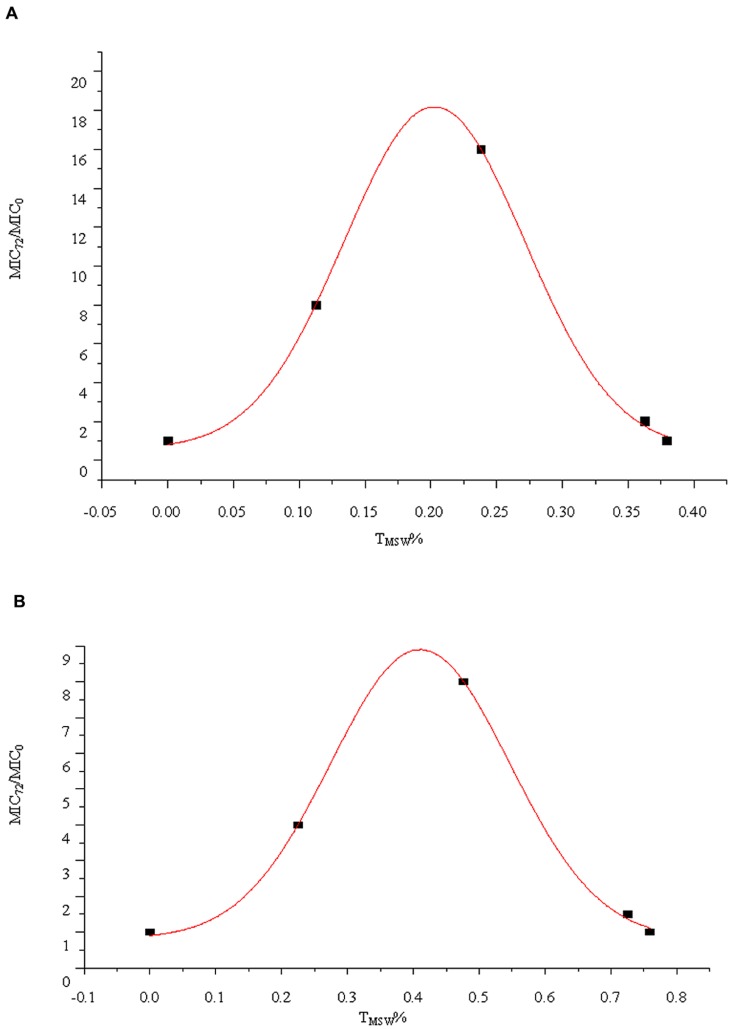
**Relationship between the T_MSW_% and MIC_72_/MIC_0_ for *S. aureus* ATCC 25293 exposed to cefquinome in the *in vitro* model with two terminal half-lives (t_1/2β_). (A)** t_1/2β_ = 3 h, **(B)** t_1/2β_ = 6 h.

According to Gaussian function and regression analysis results, the bacteria was prone to develop resistance when T_MSW_% was about 20% (x_0_ = 0.2027) in groups with t_1/2β_ of 3 h after multiple dosages (*R*^2^= 0.9989), and T_MSW_% was about 40% (x_0_ = 0.4102) in groups with t_1/2β_ of 6 h after multiple dosages (*R*^2^ = 0.9986), respectively, as shown in **Figure 5**. Those results were consistent with the data from **Table 1**.

## Discussion

Antimicrobial resistance has been a global problem and a great number of strategies have been proposed to slow the emergence of resistance (Stratton, 2003; Levy and Marshall, 2004). *In vivo* or *in vitro* PK/PD models have previously been applied to optimize the dosage regimen of various antibiotics in the literature (Croisier et al., 2004b; Ferran et al., 2009; Andraud et al., 2011; Gebru et al., 2012). However, PK/PD model has been renewably used to investigate relationship of bacterial resistance with AUC/MIC, C_max_/MIC and T > MIC, recently. In the present study, we simulated the cefquinome pharmacokinetic profiles with different terminal half-lives using this dynamic model and tried to predict the selection of resistant *S. aureus* with some indices, such as T > MIC and T_MSW_%.

It is believed that integration of drug pharmacokinetics, mutant prevention concentration is helpful in slowing the emergence of resistance. In this experiment, the MPC and MSW of cefquinome against *S. aureus* ATCC25923 were determined. The SI, ratio of MPC to MIC_99_, is the ability of a drug to select resistant mutants (Zhao and Drlica, 2001). In other word, the bigger the SI is, the easier the drug is to induce resistance. The SI of cefquinome against *S. aureus* ATCC25923 was 10.24 in our study, which suggested that cefquinome could induce *S. aureus* resistant mutation easily and effectively.

T > MIC was regarded as a promising predictor for the efficacy of cephalosporins. Previous pharmacodynamic studies have addressed separate issues around the relationships between T > MIC and efficacy for cephalosporins. Craig (1995) has reviewed the data of efficacy for cephalosporins against *Enterbacteriaceae*, *streptococci* and *S. aureus* in several animal infection models and found that the time above MIC required for a bacteriostatic effect against strains of *Enterbacteriaceae*, *Streptococci* were generally 60–70%, and 40–50% for *S. aureus*, respectively. In our study, the value of T > MIC for maximal killing was 40∼60% for cefquinome against *S. aureus* reference strain. In consistent with the widespread idea that antibacterial efficacy of β-lactams depend on T > MIC, the effect of cefquinome against *S. aureus* in our study exhibited similar correlation. As observed in **Figure 3**, killing curves of *S. aureus* exposed to each dose of cefquinome showed the typical pattern of time-dependent bactericidal action. The similar observation was also reported for other β-lactam drugs in previous studies (Lindecrona et al., 2000; Burgess and Hall, 2004). Considering that the T > MIC was one of the most important parameters for optimal dosage regimen, T > MIC was selected as a parameter to evaluate resistant mutation in this article. In the present study, the values of T > MIC ranging from 8.11 to 17.14 h were dangerous zones for inducing resistant mutation in groups with t_1/2β_ of 3 h after multiple dosages, and values ranging from 16.21 to 34.28 h were dangerous zones for inducing resistant mutation in groups with t_1/2β_ of 6 h multiple dosages. Compared these values with previously reported study data that T > MIC value ranged from 42 to 54 h for cefquinome in dairy cows (Zonca et al., 2011), the prevention of selecting resistant *S. aureus* strains seems to be achieved by the present dosage regimens approved by the European Medicines Agency (EMA) for the treatment of dairy cow mastitis.

T_MSW_ is another important parameter used for evaluating resistant mutation. A previous study conducted in a rabbit lung infection model with *Streptococcus pneumonia* showed that the selection of resistant bacteria occurred systematically when concentrations of gatifloxacin were within the MSW (T_MSW_) for more than 45% of the treatment duration (Croisier et al., 2004b). Another experiment in rabbits infected by *S. aureus* also showed that time in the MSW > 33% was preferable to select mutants (Cui et al., 2006). When the abilities of the indices T_MSW_, AUC_24__h_/MIC, C_max_/MIC to predict the selection of resistant bacteria were compared, only T_MSW_ appeared to be a good predictor of the prevention of resistance (Ferran et al., 2009). In this study, T_MSW_ was also used to predict selection of resistant *S. aureus* against cefquinome. It was clearly determined that when T_MSW_ was above 36% in groups with t_1/2β_ of 3 h after multiple dosages or above 73% in groups with t_1/2β_ of 6 h after multiple dosages, cefquinome could restrict the resistant mutation.

In the present investigation, the bacterial resistant mutation was affected by different terminal half-lives and dosages. Within the group of the same terminal half-life (3 or 6 h), the bacterial resistant mutation happened under the condition of relative low dosage (maximum concentrations were 2MIC and 4MIC). When the maximum concentration was over 8MIC, resistant mutation was prevented. In the same dosages (maximum concentration was 2MIC or 4MIC), the MIC_72__h_ increased from 8- to 16-fold in groups with t_1/2β_ of 3 h after multiple dosages and increased from 4- to 8-fold in groups with t_1/2β_ of 6 h after multiple dosages, respectively. Those results suggested that the bacterial resistant mutation might happen at a low concentration. In high dosages or long terminal half-lives, the drug concentrations might exceed the MPC value, the upper boundary of MSW, and the resistant mutation could be inhibited. The conclusion from this study was consistent with other similar studies (Cui et al., 2006; Ferran et al., 2009; Zhu et al., 2012).

The limitation of the study was that only one reference strain was used to simulate the efficacy of cefquinome in this *in vitro* model; however, the multi-resistance is becoming an urgent trend all over the world. It may be reasonable that more clinical isolates of *S. aureus* could be used for further study. Firsov et al. (2008) used two methicillin-resistant strains of *S. aureus*, ATCC 6538 and ATCC 43300, to study the enrichment of ciprofloxacin resistant mutation in an *in vitro* dynamic model. Liang et al. (2011), chose three clinical isolates of *S. aureus*, SA99, RN450 and RN450-A1, to analyze the enrichment of levofloxacin resistant mutation with *in vitro* dynamic model. From the results obtained in the dynamic model, more clinical isolates could be used to study resistant mutation easily based on the substantial method. Moreover, to our knowledge, evaluation of antimicrobial activity for cefquinome with different terminal half-lives is scarce. Considering the fact that cefquinome is the time-dependent drug, it is positive to study the correlation resistant mutation with different terminal half-life conditions. Another limitation of the current study was the absence of resistance mechanisms of *S. aureus* observed *in vitro*. Therefore, the pattern of *S. aureus* resistant to cefquinome, such as production of β-lactamases, alteration of drug targets, porin-mediated resistance, and/or eﬄux-mediated resistance, needs confirmation in the further study.

## Conclusion

The present study of resistant mutation with *S aureus* ATCC 25923 exposed to cefquinome with two different terminal half-lives in *in vitro* dynamic models supports the MSW hypothesis and provides some useful parameters to predict the resistant mutation, especially T > MIC and T_MSW_%. Further study will concentrate on more clinical isolates which could be used for verification of these results and resistance patterns of *S. aureus* resistant to cefquinome.

## Author Contributions

All authors listed, have made substantial, direct and intellectual contribution to the work, and approved it for publication.

## Conflict of Interest Statement

The authors declare that the research was conducted in the absence of any commercial or financial relationships that could be construed as a potential conflict of interest.
